# Attention to faces in images is associated with personality and psychopathology

**DOI:** 10.1371/journal.pone.0280427

**Published:** 2023-02-15

**Authors:** Marius Rubo, Ivo Käthner, Simone Munsch

**Affiliations:** 1 Cognitive Psychology, Perception and Research Methods, Department of Psychology, University of Bern, Bern, Switzerland; 2 Clinical Psychology and Psychotherapy, Department of Psychology, University of Fribourg, Fribourg, Switzerland; 3 Department of Psychology I, Biological Psychology, Clinical Psychology and Psychotherapy, University of Würzburg, Würzburg, Germany; CNRS: Centre National de la Recherche Scientifique, FRANCE

## Abstract

Humans show a robust tendency to look at faces in images, but also differ consistently in the strength of this attentional preference. Previous research remained inconclusive as to how a stronger face preference may be indicative of an individual’s personality or clinical characteristics. Here we investigated face preferences in 120 participants (primarily drawn from a student population) who freely viewed photos in an internet browser showing a person in the context of a visually rich environment while attention was assessed using a cursor-based technique. Participants differed consistently in the strength of their face preference across images. A stronger preference for faces was correlated positively with openness to experience, extraversion, agreeableness and empathizing and was correlated negatively with social anxiety, depression levels and alexithymia. Trait measures were linked through a strong common factor which was additionally correlated with face preference. We conclude that face preferences may be linked to personality traits and to psychopathology but that an attribution to a specific facet of psychopathology may not be warranted. Future research should investigate links between face preferences and personality features in more diverse samples and across differing social situations.

## Introduction

Human attention is robustly captured by faces [1, 2]. A tendency to attend to faces can be observed from a very young age in human [3, 4] as well as nonhuman [5] infants and provides a basis for every higher order social capability [6]. Attentional preference for faces was found to robustly vary between observers, with some people consistently allocating more time to attending to faces than others [7–9]. However, data on the significance of this attentional trait for psychological functioning remain fragmented. Different lines of research investigated relationships between face preference and personality traits as well as psychological constructs derived from clinical and social psychology, with overall inconclusive results.

Using the Big Five personality traits consisting of extraversion, agreeableness, conscientiousness, neuroticism, and openness to experience, [10] found a positive correlation between face preference and extraversion as well as agreeableness and a negative correlation with openness, whereas [9] found only a negative correlation between face preference and conscientiousness and [8] found no correlations between face preference and any Big Five personality trait. A prediction of personality traits from behavioral measures are of particular interest as they provide a parsimonious description of inter-individual differences which is widely used in social and clinical psychology [11, 12] and generalizes well across cultures [13].

Other studies have linked attentional preferences in social situations to clinical descriptions of inter-individual variation. Social anxiety, a fear of social situations which is common in the general population [14] and, when pronounced and debilitating, can justify a diagnosis as social anxiety disorder (SAD) [15], was linked to eye contact avoidance in clinical reports [16]. Investigations of gaze towards social images, however, yielded mixed results with studies reporting a negative [17, 18], positive [19, 20] or no [9, 21] association with face preference. Enhanced sensitivity to social rejection, which is found in SAD, but also in major depression and other mental disorders [22], was associated with faster attention towards rejecting faces [23, 24] as well as sad faces [25]. Trait anxiety was found to be associated with an attentional bias towards threat, including threatening faces [26, 27]. Depression levels were found to be associated with an attentional bias towards negative information, including sad faces [28, 29]. Disordered eating was reported to be associated with an attentional bias towards faces in general [30], but also to rejecting faces [31]. Individuals with Autism Spectrum Disorder (ASD) are known to avoid eye contact in real social encounters [32, 33] and were also found to exhibit reduced attention towards faces in images [34, 35], although findings are not entirely consistent [36] and more mixed with regards to autistic traits [9, 37, 38]. A contrasting juxtaposition between *empathizing* (the ability to recognize mental states in others and respond emotionally) and *systemizing* (the drive to analyze rule-based systems) is used to describe a variation in human mindsets on a scale which seamlessly transitions from the normal population to individuals with ASD [39]. Other constructs used to describe differences between people can be assumed to be associated with a tendency for face preference. Alexithymia, the difficulties to identifying, differentiating and describing feelings [40], was found to be associated with low social functioning [41] and to predict poorer recognition of emotional expressions in faces [42]. Impulsivity may influence face preferences due to its association with inattention [43]. Social value orientation (SVO), the magnitude of concern people have for others [44], predicts cooperation in social dilemmas [45] and may be reflected in the seeking of social information, although a previous study found no correlation with attention to faces [9]. Since general knowledge may reflect aspects of an individual’s personality [46, 47], it may likewise be reflected in attentional preferences.

It may be noted that attentional preferences in social situations were frequently linked to isolated psychological constructs, although psychological traits are often interrelated. Such interrelations were observed between personality and clinical characteristics [48, 49] and in particular among different clinical characteristics, where the observation of a strong common factor (general psychopathology, or *p*) led [50] to suggest that any finding with regard to a specific form of psychopathology should be taken to generally imply a relationship with *p* unless specificity can be established (Note that this rationale represents a renunciation from a scientific practice where claims for generality but not specificity are particularly scrutinized). To this end, the present study assessed a range of personality and clinical characteristics, allowing to identify relationships both with individual variables (possibly establishing specificity) and their shared variance in a student population.

Various methods exist to assess a preferential processing of faces. While some studies measured reactivity to external cues in the presence of social stimuli [24, 31], others obtained gaze behavior in response to seeing faces [19, 25]. An increasing number of studies recorded gaze behavior in response to more naturalistic stimuli which encompass the complexity of everyday experiences rather than showing faces in isolation [e.g., 2, 9, 35]. In the present study, we additionally selected stimuli based on the presence of non-social areas which may draw attention away from faces, avoiding a situation where participants may attend to faces partly in response to a lack of other interesting areas to visually explore. We used a measurement technique where participants reveal their attentional preferences by moving a computer mouse to unblur individual areas in an otherwise blurred image [51–54], a technique which was found to approximate eye fixations towards unblurred images both when unblurring occurred continuously while participants were moving their cursors [52] and when participants had to click to unblur an area in the image [53]. This form of attentional assessment can be employed in internet-based experiments, allowing to reach wider and more diverse samples of participants while often preserving findings observed with conventional methods across a range of experimental tasks [52, 55].

The present study assessed exploratively if attentional preferences for faces were associated with several influential psychological traits. We first inspected if attention towards images in a web browser exhibited two patterns commonly observed in eye-tracking studies: a high intra-individual consistency in face preferences across images [7–9] and a stronger face preference towards direct compared to averted gaze [56]. We then compared participants’ gaze preferences with their personality traits, social anxiety, rejection sensitivity, trait anxiety, depression levels, eating psychopathology, systemizing and empathizing, alexithymia, impulsivity, social value orientation and general knowledge. We additionally assessed if variance shared between these constructs may represent a more parsimonious explanation of observed effects.

## Materials and methods

### Subjects, materials and procedure

120 participants (100 women, 18 men, 2 persons who did not wish to disclose or did not identify with one of the two genders; mean age = 22.05 years, *SD* = 5.92, 110 university students, 81 living in Germany and 39 living in Switzerland) took part in this study. This study’s total sample size and obtained measures were preregistered (AsPredicted #55857, https://aspredicted.org/EZJ_ZVA). The data that support the findings of this study are available at https://osf.io/5gvwb/. Original images are available online (for weblinks see Supplementary Methods 1.1 in S1 File). Cut images, region-of-interest maps and analysis code for this study are available by emailing the corresponding author. A sample size of n = 120 is sufficient to detect a correlation of *r* = .25 with a Power of 80% and Alpha set to 5% in a two-sided comparison. Before valid datasets from 120 participants were obtained, we recorded data from an additional 6 participants which were excluded due to missing mouse movements in at least one trial. The study conformed to the principles expressed in the Declaration of Helsinki and was approved by the local ethics committee at the University of Fribourg (Ref-No. 2020–661R1). Participants entered the study through a weblink. They were informed that they could use a computer with a mouse (used by 44 participants) or a trackpad (used by 76 participants), but not a smartphone or tablet.

After completing an informed consent form and a demographic questionnaire, participants viewed 20 images for 10 seconds each in a randomized order. All images depicted one person with clearly visible face, with people in 10 images looking directly into the camera (direct gaze) and people in the remaining 10 images looking elsewhere (averted gaze). All images were awarded by the Wikimedia Commons community (https://commons.wikimedia.org/) for their visual appeal and depicted a visually rich context (e.g., a store, a backyard, a garage). Images were blurred and only displayed at high acuity around the cursor position. Image resolution was 1280 × 720 pixels. The blur effect was achieved using a Gaussian Blur filter with a radius of 20 pixels. The image area displayed at high acuity was defined by a circular mask with a diameter of 144 pixels. Mouse cursor positions were assessed continuously (recording *M* = 22.50 (*SD* = 1.72) positions per second).

### Self-report measures

Subsequently, participants filled out questionnaires assessing the Big Five Personality traits, Social Anxiety, Rejection Sensitivity, Trait Anxiety, Depression levels, Eating Psychopathology, Systemizing, Empathizing, Alexithymia, Impulsivity, Social Value Orientation and General Knowledge.

**Personality Traits** were assessed using the German version of the 21-items short form of the Big Five Inventory which include extraversion, agreeableness, conscientiousness, neuroticism, and openness to experience (BFI-K; [57]). Items ask for the agreement with statements on perceptions about oneself and are scored from 1 (strongly disagree) to 5 (strongly agree).

**Social Anxiety** levels were assessed using the German translation [58] of a short version of the Social Interaction Anxiety Scale (SIAS-6; [59]). The SIAS-6 is a 6-item shortened versions of the 20-item original scale. Items are rated on a 5-point Likert scale (0 = Not at all characteristic or true of me, 4 = Extremely characteristic or true of me). Correlations with the long version [60] were reported to be high (r = .88; [59]).

**Rejection Sensitivity** was assessed using a German translation of the Adult Rejection Sensitivity Questionnaire (A-RSQ; [61]). Participants read a brief description of nine hypothetical situations in which rejection by a significant other is possible (e.g., “After a bitter argument, you call or approach your significant other because you want to make up.”). Participants are asked to indicate the degree of their concern or anxiety about the outcome of each situation on a 6-point scale ranging from 1 (very unconcerned) to 6 (very concerned). Rejection sensitivity is scored by multiplying the level of concern/anxiety by the reverse of the level of acceptance expectancy.

**Trait Anxiety** was assessed using a the German translation of the short version of the trait part of the State-Trait Anxiety Inventory (STAI-T; [62, 63]). The 10 items ask for agreement on an eight-level Likert scale ranging from ‘not at all’ to ‘absolutely’.

**Depression levels** were assessed using the German version of the Patient Health Questionnaire (PHQ-9; [64, 65]) which consists of nine items. Participants are asked to report on the presence of nine problems in the last 2 weeks on a 4-point scale ranging from “not at all” (0 points) to “nearly every day” (3 points). The scores for symptom severity are 5–9 for mild, 10–14 for moderate, 15–19 for moderately severe and 20–27 for severe.

**Eating Psychopathology** was assessed using a brief German version of the Eating Disorder Examination Questionnaire (EDE-Q8; [66]). The 8 Items ask participants to rate various aspects of eating psychopathology on a 7-point scale (ranging from “1—no day” to “7—every day”) for the past 28 days.

**Systemizing and Empathizing** were assessed using German translations of the Empathy Quotient and the Systemizing Quotient (EQ-10 and SQ-R-10; [39]) which consist of ten items each. The questionnaires have conceptual overlap with those used to assess autistic traits in previous studies [67], show a wide distribution in the normal population [39] and are collectively highly predictive of autistic traits [39]. An additional six questions which were exploratively posed as alternative measure for a preferred focus on social information (see pre-registration) were not analyzed further due low consistency (*α* = .20).

**Alexithymia** was assessed using the German version of the Toronto Alexithymia Scale (Toronto Alexithymia Scale; TAS-20; [68, 69]). The scale consists of 20 items and was proposed to consist of three subscales (difficulty identifying feelings; externally orientated thinking; importance of emotional introspection), but due to a lack of empirical support for a three-factor structure [69, 70], the present study instead employs the scale’s total score.

**Impulsivity** was assessed using a short German version of the Barratt Impulsiveness Scale (BIS-15; [71, 72]). The 15 items are grouped in the three factors nonplanning (e.g., ‘I plan tasks carefully (inverted).’), motor (e.g., ‘I say things without thinking.’), and attentional (e.g., ‘I get easily bored when solving thought problems.’) impulsivity. Items are answered on a four-point scale ranging from rarely/never to almost always/always. Scores of the short form are highly correlated with the full version (r = 0.94; [72]).

**Social Value Orientation** was assessed using the six primary resource allocation decisions in the eponymous measure (SVO; [44]). Here, SVO is conceptualized as as the weights people assign to their own and to others’ outcomes in a distribution task. Participants were asked to allocate money to a hypothetical other and to themselves in a situation characterized by reciprocal dependency, the more money participants allocated to oneself, the less the other person would receive and vice versa. SVO scores were computed as inverse tangent of the ratio of the mean allocation to oneself and to the other person. A higher SVO angle indicates a prosocial or even altruistic orientation, while a smaller SVO angle indicates more individualistic or competitive orientation. Internal consistency in the present study was computed after applying the same trigonometric operation on all individual items instead of their sum.

**General Knowledge** was assessed using 40 questions from a larger assessment battery used in [73] covering a range of topics (history, economy, culture and science) and to not require knowledge which may be common only for the time of publication of the original question set. In each question, participants were asked to identify the correct answer among four possible answers (resulting in a chance level of 25%).

Questionnaire descriptives are shown in Table 1. Data from six questionnaires (SIAS, RSQ, STAI, PHQ, EDEQ, Systemizing) showed a right-skewed distribution and were log(x+1)-transformed for the purpose of statistical analyses, while descriptive statistics report untransformed data.

**Table 1:** Sample questionnaire descriptives.

	Mean	SD	Range	Possible Range	*α*
Extraversion	3.3	0.86	[1.5, 5]	[1, 5]	0.81
Agreeableness	3.3	0.73	[1.25, 4.75]	[1, 5]	0.62
Conscientiousness	3.7	0.72	[1.75, 5]	[1, 5]	0.78
Neuroticism	3.3	0.93	[1, 5]	[1, 5]	0.83
Openness	4.11	0.67	[2.4, 5]	[1, 5]	0.75
Social Anxiety	4.91	4.38	[0, 21]	[0, 24]	0.85
Rejection Sensitivity	10.03	4.15	[2.78, 25.67]	[1, 36]	0.74
Trait Anxiety	16.11	7.48	[0, 34.29]	[0, 100]	0.85
Depression levels	7.33	4.61	[1, 26]	[0, 27]	0.84
Eating Psychopathology	1.64	1.52	[0, 6]	[0, 6]	0.93
Systemizing	5.55	3.39	[0, 17]	[0, 20]	0.67
Empathizing	11.79	4.03	[0, 20]	[0, 20]	0.82
Alexithymia	46.3	11.11	[21, 87]	[20, 100]	0.87
Impulsivity	30.98	6.09	[19, 48]	[15, 60]	0.53
Social Value Orientation	38.5	4.47	[23.2, 46.42]	[20.17, 51.79]	0.58
General Knowledge	58.92	12.44	[32.5, 82.5]	[0, 100]	0.71

Range: Actual range of scores in the present sample. Possible range: Minimum and maximum scores that can be reached on each measure. *α*: Cronbach’s alpha.

All levels and variations on measures used to assess psychopathology were comparable with other samples drawn from the general population. Previous observations include *M* = 5.99 (*SD* = 4.67) with n = 72 for social anxiety [74], *M* = 9.46 (*SD* = 4.76) with n = 347 for rejection sensitivity [75], *M* = 17.49 (*SD* = 4.39) with n = 1141 for trait anxiety [76], *M* = 3.30 (*SD* = 3.65) with n = 2693 for depression levels [76], *M* = 0.94 (*SD* = 1.14) with n = 4360 for eating psychopathology [66], *M* = 6.09 (*SD* = 4.13) with n = 634955 for systemizing [39], *M* = 9.83 (*SD* = 4.98) with n = 634955 for empathizing [39], *M* = 50.07 (*SD* = 11.06) with n = 965 for alexithymia [68] and *M* = 30.04 (*SD* = 6.13) with n = 752 for impulsivity [71]. When studies reported questionnaire data separated by gender, we pooled data across gender categories with pooled variance defined as the mean of the variances plus the variance of the means of each data set [77].

### Data processing

Attention in each sample was labelled to rest either on a face (if at least part of the face was made visible at a high acuity) or elsewhere. The temporal trajectory of attention showed a distinct pattern, with more time spent attending to a face during the first 5 seconds compared to the last 5 seconds (see Supplementary Results 2.1 in S1 File). Mouse movements were generally present throughout the time of stimulus presentation, with a peak in the beginning (see Supplementary Results 2.2 in S1 File). Attention data were aggregated as percentage of time spent attending the face across the whole trial. Since data showed a right-skewed distribution (see Supplementary Results 2.3 in S1 File), they were log(x+1)-transformed for the purpose of statistical analyses (while descriptive statistics and plots show untransformed data). Attention data were further averaged within participants and conditions (when comparing attention between conditions) or only within participants (when describing correlations between attention across all images and questionnaire data).

### Data analysis

Data were analyzed using R for statistical computing [version 3.2, 78]. Internal consistency of measures was assessed with Cronbach’s *α*. Time spent attending to faces was predicted by the condition (direct versus averted gaze) in a linear mixed model which included a random intercept for each participant. Parameters were tested for significance using an *F*-test. Cohen’s *d* was defined as the mean difference between the two conditions divided by the standard deviation of the difference. Relationships between attention to faces and questionnaire data were tested using Pearson correlations. P values were adjusted for multiple comparisons following a procedure described in [79]. Principal component analysis (PCA) was performed with the function *princomp* with no rotation applied. In all analyses, *α* was set to 5%. Normality was inspected using Q-Q Plots.

Among the variables which were significantly correlated with attentional preferences towards faces, we employed a hierarchical data analysis strategy to test if personality factors alone allowed for the best prediction, or if any other questionnaire data exhibited predictive power above and beyond what was explained by personality factors. To this end we first performed an all-subsets multiple regression analyses including every combination of personality factors which were significantly correlated with attentional preferences towards faces. Model performance was evaluated by means of the RMSE criterion across leave-one-out cross-validated models (with data from one participant omitted from the training data set and used as sole test data set in each iteration). Next, all other variables which were significantly correlated with attentional preferences were added to the best-performing model from the first step in a second all-subsets multiple regression analyses.

## Results

### General properties of attention to faces

**Fig 1:**
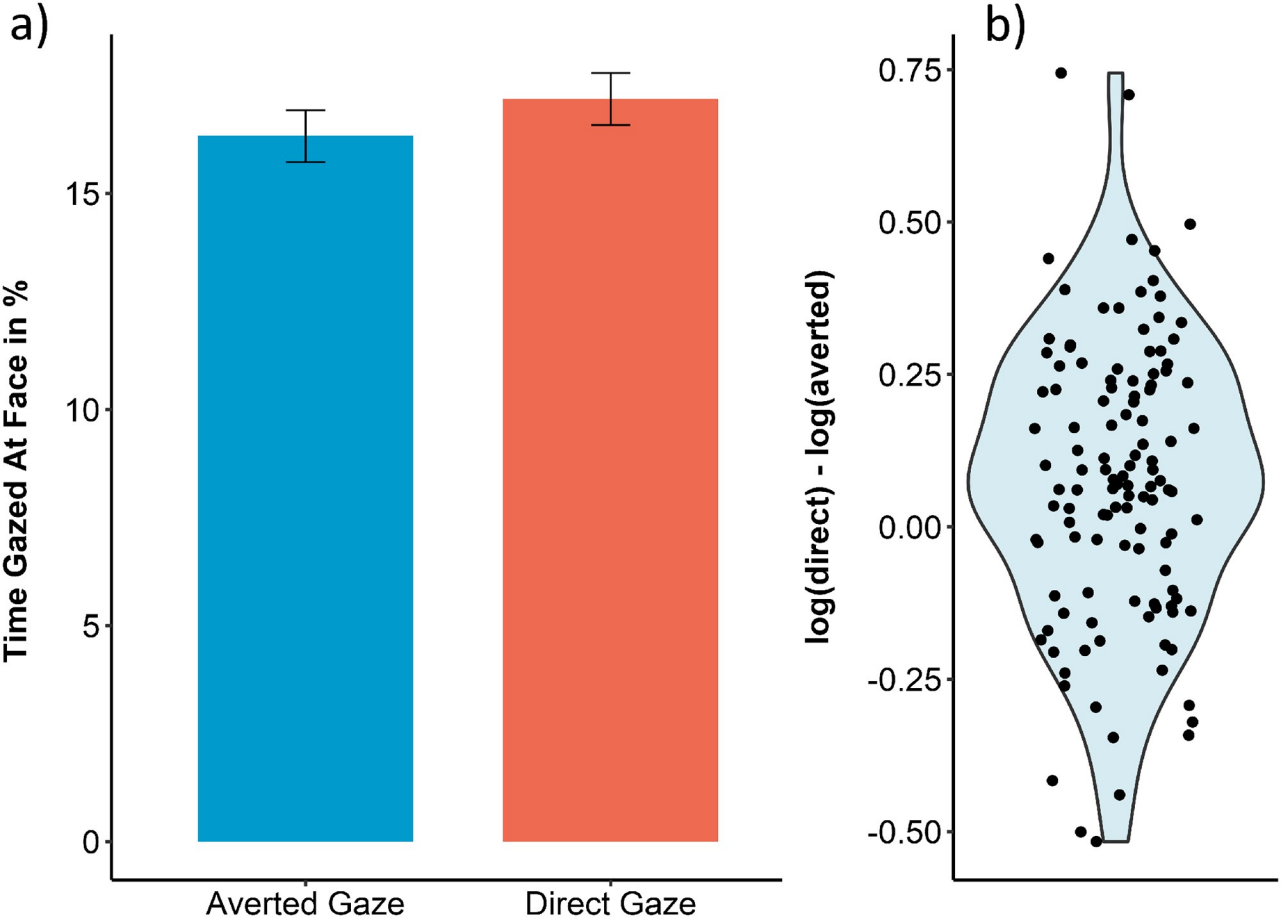
(a) Time spent looking at faces when they looked away from the camera (averted gaze) compared to when they looked into the camera (direct gaze). Error bars represent SEM. (b) Violin plot showing the differences between attention towards faces when depiciting direct compared to averted gaze for all 120 participants. Positions on the x-axis are randomized for better visibility. This graph shows log-transformed data, directly corresponding to the statistical analysis described in the text.

Participants spent an average of 16.76% (*SD* = 6.21%, Range = [5.96 44.08]) of the image viewing time attending to faces. They attended to faces depicting direct gaze for longer (*M* = 17.19%, *SD* = 6.61%, Range = [5.14 41.12]) compared to faces depicting averted gaze (*M* = 16.33%, *SD* = 6.58%, Range = [5.61 47.03]; F(1,119) = 10.99, *p* = .001, *d* = 0.30 [95%-CI = 0.12, 0.49]; see Fig 1). Internal consistency for attention towards faces was *α* = 0.88 across all images, *α* = 0.80 across images depicting direct gaze and *α* = 0.76 across images depicting averted gaze.

### Correlations between self-reports and attention to faces

**Fig 2:**
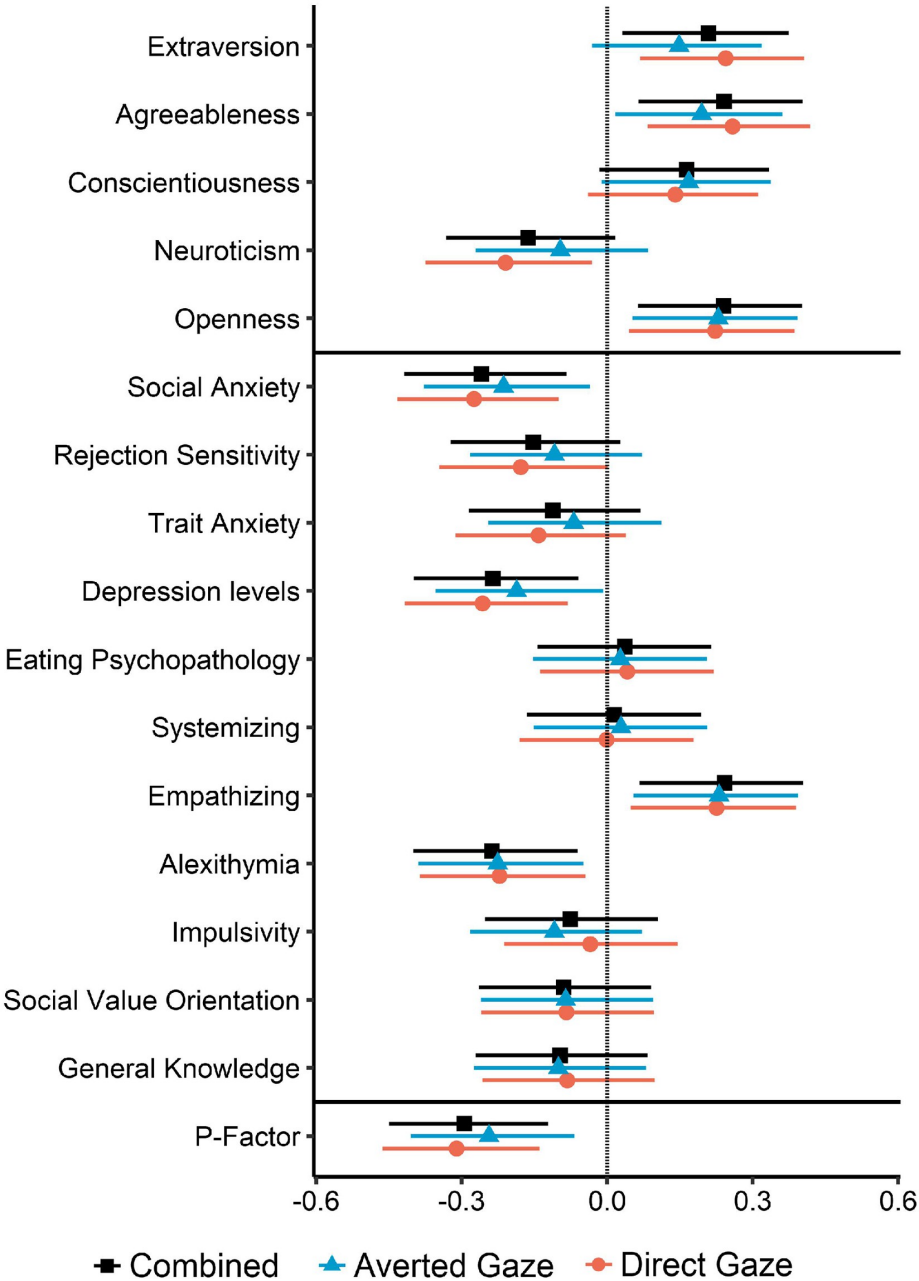
Correlations between attention towards faces and personality measures (above the first horizontal line), several clinical and other psychological measures as well as a general psychopathology factor (*p*-factor, lowest line). Results are presented for attention to faces across all 20 images as well as for only the 10 images depicting averted and direct gaze, respectively. Error bars represent 95%-CIs.

Across all images, attention to faces was positively correlated with extraversion, agreeableness, openness to experience and empathizing and negatively correlated with social anxiety, depression levels and alexithymia (see Fig 2 and Supplementary Results 2.4 in S1 File for details). Correlation coefficients in the two sets of images depicting direct and averted gaze were highly consistent (r = 0.96, 95% CI [0.88, 0.99], t(14) = 12.60, *p* <.001).

### Factor structure among trait measures

**Fig 3:**
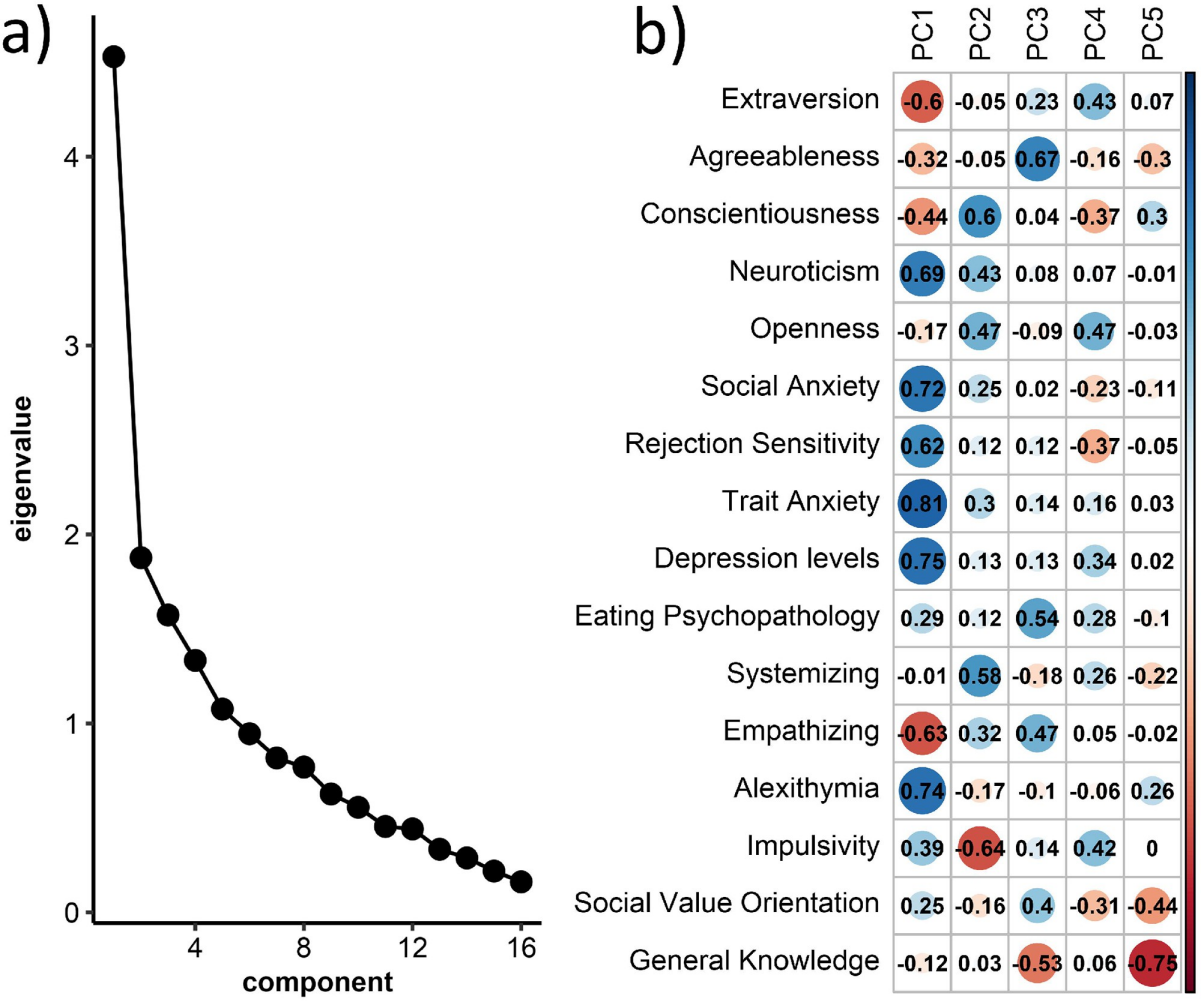
Results of a principal component analysis (PCA). (a) shows the factors’ eigenvalues in a scree plot, while (b) shows correlations between the first five factors and each variable.

We investigated the factor structure among all trait variables using Principal component analysis (PCA). Based on a scree plot (Fig 3a), we identified one dominant factor which explained 28.31% of the total variance. (Fig 3b) shows the correlations between each variable and the five strongest factors (which yielded eigenvalues of at least 1). Due to its overall positive correlation with variables linked to psychopathology, we refer to the first factor as *p*-factor or *p* [50]. P was negatively correlated with attention to faces (r = -0.29, 95% CI [-0.45, -0.12], t(118) = -3.35, *p* = .019).

### Predicting attention to faces beyond personality traits

In a hierarchical analysis (for details see Supplementary Results 2.5 in S1 File), we assessed if any variable predicted attention towards faces above and beyond what was explained by personality factors alone (a particularly widely used and parsimonious characterization of differences between individuals). In an all-subsets multiple regression analyses using the three personality factors which correlated significantly with attention towards faces (extraversion, agreeableness and openness), the best-performing model incorporated only agreeableness (*β* = 0.25 [95%-CI 0.08, 0.42]) and openness (*β* = 0.25 [95%-CI 0.08, 0.42]). A second all-subsets multiple regression analyses adding all other variables which were correlated significantly with attention towards faces (social anxiety, depression levels, empathizing and alexithymia) found that only the inclusion of social anxiety (*β* = -0.22 [95%-CI -0.39, -0.05]) led to an additional improvement in the model’s predictive accuracy.

## Discussion

Previous research observed consistent differences in people’s attentional preference for faces but remained ambiguous towards the possibility to infer psychological traits from this behavioral marker. We found that attention to faces was positively associated with openness to experience, extraversion, agreeableness and empathizing while being negatively associated with social anxiety, depression levels and alexithymia.

Both a positive correlation between attention towards faces and extraversion as well as agreeableness was reported before [10], although other studies did not observe this effect [8, 9] and, unlike the present study, a previous study observed a negative correlation between attention towards faces and openness to experience [10]. A negative correlation between attention to faces and social anxiety corresponds to clinical reports of SAD [16], although previous studies did not consistently observe this effect during the viewing of static images [9, 17–21]. Negative correlations between attention towards faces and depression levels [which were previously found to correlate with attention towards sad faces; [29]] and alexithymia [which is associated with poorer recognition of emotional expressions in faces; [41, 42] have no direct equivalent in the literature, but may be seen to generally comply with previously observed patterns. Although the present study did not observe a correlation between attention towards faces and systemizing—which would correspond to previous observations of reduced face preference in ASD and autistic traits [32, 34, 35] but was similarly not observed by [9]—the positive correlation between attention towards faces and empathizing (which is characterized as a counterpart to autistic traits) may imply a similar notion.

With correlations between attentional preferences towards faces and several psychological measures, attentional preferences in the present study cannot be directly linked to a specific characteristic (e.g., dissociate if a person’s reduced preference for faces is more likely to reflect low levels of extraversion or high levels of social anxiety). This relative lack of specificity is further reflected in the presence of a strong common factor among the assessed variables. As this factor was positively correlated with the assessed clinical characteristics of social anxiety, rejection sensitivity, trait anxiety, depression levels, alexithymia and impulsivity, we argue that it may best be described as representing general psychopathology, or *p* [50, 80]. Its positive correlations with neuroticism, which was observed to be correlated with social anxiety [48], and its negative correlation with extraversion, which was found to be negatively correlated with depression levels [49], may corroborate this notion. As several of its correlates, *p* was negatively correlated with attentional preferences for faces. Among the seven variables which showed a statistically significant correlation with attentional preferences towards faces, only agreeableness and openness did not load strongest on *p*, indicating a partial separability between personality traits and *p* in the present sample.

Since *p* represents a latent variable and cannot be directly assessed, a practical question concerns the most parsimonious assessment of data which explain variance in face preference. The present finding that social anxiety levels stood out as only variable to explain face preference above and beyond a model consisting of personality factors highlights that this variable may hold practical utility in explaining and understanding face preferences.

Attention patterns towards images in an internet browser were overall comparable with data from eye-tracking studies, with consistent differences in face preferences across images (*α* = 0.88) and a stronger tendency to attend to faces showing direct gaze (although due to our use of naturalistic images we cannot rule out that uncontrolled factors such as facial properties, lighting conditions or background objects contributed to differences in face preferences between images). This points towards the validity of this method to generally approximate eye fixations as observed by [52, 53], but it may be noted that important differences between gaze-based and cursor-based attention assessment may nonetheless exist. Participants’ reactions in cursor-based attention techniques are typically slower compared to their gaze behavior [51, 54], where a first saccade towards relevant stimuli in an image can often be observed within just 300ms [81]. Cursor-based assessments of attention may thus not be suitable when studying the earliest attentional reactions to stimuli. Moreover, gaze was frequently observed to be attracted by image regions with high low-level physical saliency [e.g., contrast, luminance, color; [2, 7, 82, 83]. While this effect was observed to be preserved when participants viewed blurred images [84], it is unclear to what extent cursor-based attention techniques similarly capture saliency-driven attention. More generally, with complex coordination between gaze and hand movements, cursor-based assessment may more strongly capture top-down as opposed to bottom-up processes [85].

Time spent attending to faces was relatively low at an average of 17% of stimulus presentation time (and 29% in the first 3 seconds of stimulus presentation) compared with 55% in a study by [9] (where stimuli were presented for 3 seconds). This difference may result from a different assessment technique, but could also be due to the selection of images which show a rich environment compared with more inconspicuous backgrounds used in several images in previous studies [2, 8, 9]. Future research should resolve if visually rich environments draw attention away from faces and if this process is modulated by psychological traits. Specifically, it may be tested if the choice of stimulus material (more complex and naturalistic images compared to more minimalistic images) influences correlations between attentional preferences and psychological traits.

### Limitations

Several constraints on the generality of the presented observations are noteworthy. Firstly, while the consistency of face preferences was observed in various sets of relatively unstandardized images and videos [7–9], generalizability towards other environments and specifically towards real-life situations may be limited. Several researchers have noted that real social encounters differ qualitatively from the viewing of images in that they provide opportunities for (and possibly fear of) social interactions [86, 87]. In real-life situations, a tendency to attend to faces was found to be weaker [88, 89] and to be more strongly influenced by social anxiety [21].

Secondly, participants in the present study were predominantly drawn from a student population in Germany and Switzerland and we did not collect data regarding racial or ethnic identification. Future studies should investigate in other samples if findings generalize towards more randomly drawn samples within the Western world and beyond [90]. The sample futhermore consisted predominantly of women. Future research should therefore investigate if gender alone (rather than personality traits or psychological characteristics) better explain differences in viewing preferences such as the more exploratory scanning strategy found in women [91]. Additionally, while face preference was found to discriminate between individuals differing in lower to medium ranges of psychopathology, it remains unclear if a discrimination of individuals differing in higher ranges of psychopathology is similarly possible.

While we refer to the observed strong factor among employed variables as *p*, one may speculate if reduced face preferences can be more specifically linked to specific forms of psychopathology. For instance, it may appear plausible to more specifically characterize the dominant first factor among assessed variables as *internalizing*, a transdiagnostic factor which includes depressiveness, anxiety and social anxiety [92]. However, the factor observed here does not perfectly fit with internalizing and may partly overlap with its counterpart, *externalizing*, which accounts, among others, for antisociality. Alternatively, the dominant factor could largely represent social aversion since extraversion, agreeableness, social anxiety and empathizing directly refer to the processing of social situations and depressiveness and alexithymia can contain elements of impaired social functioning. However, note that impairments in social functioning are not specific for individual forms of psychopathology but are found in a variety of mental disorders [93, 94]. For this reason, we followed a recommendation expressed in [50] to assert that our observation relates to *p* until a more specific characterization can be justified. Future research may employ an even broader range of self-report measures to more comprehensively assess if psychopathology, internalizing, social aversion or a different construct best accounts for reduced face preferences.

Note also that more generally, with high correlations between different correlates of face preference, we can in principle not rule out that only some of them, (or only one, or even none of them) are actually causally linked to face preference, while all other links represent merely spurious correlations. We would argue, however, that the observation that no individual construct was more strongly correlated with face preference than *p* speaks to the validity of general psychopathology as a construct which may well explain other phenomena such as attentional biases. Future research could additionally investigate more closely if individual aspects of psychopathology play a unique role in eliciting alterations in face preference by testing patient populations where, although co-morbidity represents the rule rather than the exception, one aspect of psychopathology is often pronounced. Researchers could furthermore more closely track the trajectory of face preferences in comparison to the emergence of psychopathology. For instance, if the onset of social anxiety was associated with a reduced face preference, but a later additional onset of depressive symptoms [95] was not associated with an additional reduction in face preference, one could attribute a unique causal relationship between social anxiety and a reduction in face preference.

### Conclusion

We found robust inter-individual differences in face preference which were positively associated with openness to experience, extraversion, agreeableness and empathizing as well as negatively associated with social anxiety, depression levels and alexithymia. The pattern of results is not in line with the interrelation of face preferences with any specific psychological characteristic, but rather with the shared variance between characteristics which may most parsimoniously described as general psychopathology. The overlap between personality constructs and clinical characteristics within the same factor corroborates not only the dimensionality of psychopathology [96], but more specifically the notion that characterizations of dysfunctional personality features should more strongly connect with broader conceptualizations of personality [97]. Behavioral markers such as face preference may inform and help to structure the establishment of such conceptualizations.

## Supporting information

S1 File Contains all the supporting tables and figs. (PDF)
