# Supplementary material for: Attention to faces in images is associated with personality and psychopathology
Source: PLoS One. 2023 Feb 15;18(2):e0280427. doi: 10.1371/journal.pone.0280427 (PMC9931157; doi:10.1371/journal.pone.0280427)
Supplement: S1 File — (PDF) [file pone.0280427.s001.pdf]

Supplementary to: Attention to Faces in Images is associated with Personality and Psychopathology

Marius Rubo
Cognitive Psychology, Perception and Research Methods
University of Bern
Bern, Switzerland
marius.rubo@unibe.ch

Ivo Käthner
Department of Psychology I, Biological Psychology, Clinical Psychology and Psychotherapy
University of Würzburg
Würzburg, Germany
ivo.käthner@uni-wuerzburg.de

Simone Munsch
Clinical Psychology and Psychotherapy
University of Fribourg
Fribourg, Switzerland
simone.munsch@unifr.ch

1 Supplementary Methods

1.1 Weblinks to images

Table S1 lists the weblinks to the images used in this study. Some of the images were cut and resized for the use of the present study. These modified versions of the original images are available upon request.

Table S1: Weblinks to the images used in this study.

Images with Direct Gaze	
1	https://commons.wikimedia.org/wiki/File:Kawah-Ijen_Indonesia_Ijen-Sulfur-Miner-01.jpg
2	https://commons.wikimedia.org/wiki/File:Tachileik_Myanmar_Kayan-People-Woman-03.jpg
3	https://commons.wikimedia.org/wiki/File:Petrol_station_in_Bénin.jpg
4	https://commons.wikimedia.org/wiki/File:Young_vendor_in_a_grocery_store_in_Don_Som_(2).jpg
5	https://commons.wikimedia.org/wiki/File:India_-_Varanasi_paper_bag_maker_-_0078.jpg
6	https://commons.wikimedia.org/wiki/File:India_-_Kolkata_electricity_meters_-_3832.jpg
7	https://commons.wikimedia.org/wiki/File:Hue_Vietnam_Citadel-of-Hu-21.jpg
8	https://commons.wikimedia.org/wiki/File:Comercio_en_la_plaza_del_9_de_abril_de_1947,_Tánger,_Marruecos,_2015-12-11,_DD_77.JPG
9	https://commons.wikimedia.org/wiki/File:The_water_transfer.jpg
10	https://commons.wikimedia.org/wiki/File:Hualien_Taiwan_Farmer-with-his-water-buffalo-01.jpg
Images with Averted Gaze	
1	https://commons.wikimedia.org/wiki/File:04-09-12-Schaupflügen-Fahrenwalde-RalfR-IMG_1232.jpg
2	https://commons.wikimedia.org/wiki/File:20191205_Haveli_Khazanchi,_Gali_Khazanchi,_Old_Delhi_0632_6743.jpg
3	https://commons.wikimedia.org/wiki/File:Seller,_rubber_hoops_004.JPG
4	https://commons.wikimedia.org/wiki/File:20160805_-_Inle_Lake,_Myanmar_-_8453_DxO.jpg
5	https://commons.wikimedia.org/wiki/File:Boy_plowing_with_a_tractor_at_sunset_in_Don_Det,_Laos.jpg
6	https://commons.wikimedia.org/wiki/File:Old_Woman_of_Bhaktapur_-_Kathmandu.jpg
7	https://commons.wikimedia.org/wiki/File:Fishing_boy_in_Laos.jpg
8	https://commons.wikimedia.org/wiki/File:Horse_shrimpers_of_Oostduinkerke_(DSCF9740).jpg
9	https://commons.wikimedia.org/wiki/File:Porto_July_2009-8.jpg
10	https://commons.wikimedia.org/wiki/File:Reading_a_newspaper._Catania,_Italy.jpg

2 Supplementary Results

2.1 Attention towards faces along the time of stimulus presentation

To investigate the temporal trajectory of attention towards faces, the percentage of time attended to faces was averaged within time bins of 500ms duration across images within each participant. Across all participants, the temporal trajectory of attention towards faces showed a distinct pattern, with more attention allocated towards faces in the first compared to the second half during each image presentation (see Figure S1). While participants attended to faces during 16.76% (SD = 6.21%) of the time across the whole image presentation time, attention allocation towards faces was 29.04% (SD = 8.96%) in the first three seconds, 22.94% (SD = 7.19%) in the first five seconds and 10.70% (SD = 8.04%) in the last five seconds.

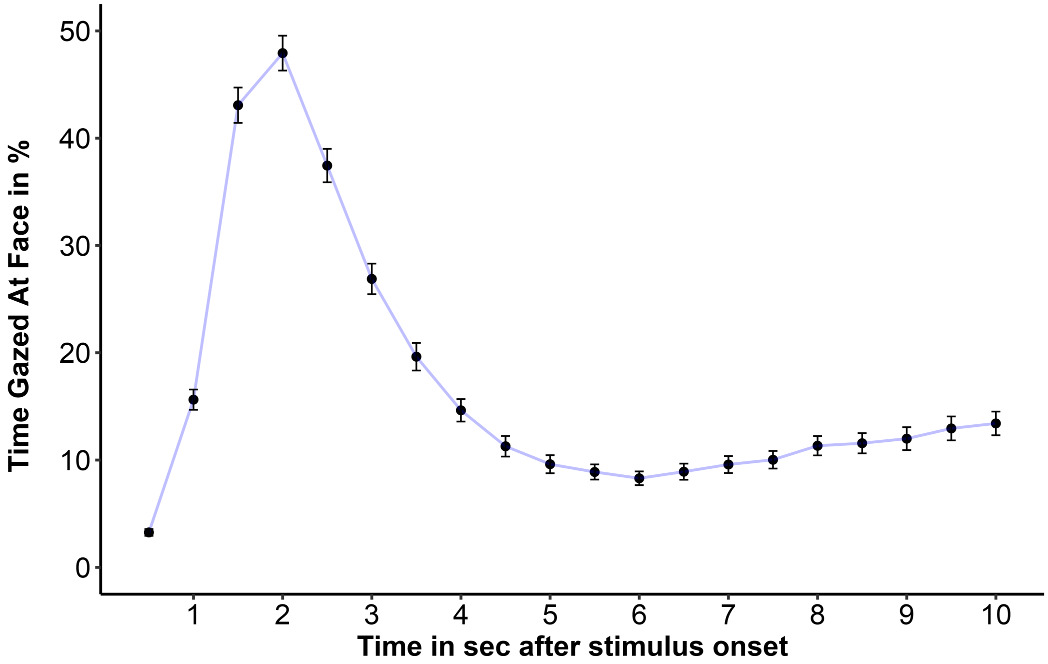

Figure S1: Temporal trajectory of attention towards faces throughout the 10 seconds of image presentation. Standard errors represent SEM.

2.2 Mouse cursor movements along the time of stimulus presentation

To illustrate the level of mouse cursor movement and its temporal trajectory throughout the time of stimulus presentation, mouse cursor speed in pixels per second was averaged within time bins of 500ms duration across images within each participant (see Figure S2). Average mouse movement speed was $M = 356.77$ pixels per second ($SD = 147.07$). The first 500ms were characterized by the smallest amount of mouse cursor movement ($M = 83.47$, $SD = 93.11$), while the second 500ms were characterized by the largest amount of mouse cursor movement ($M = 475.62$, $SD = 249.10$).

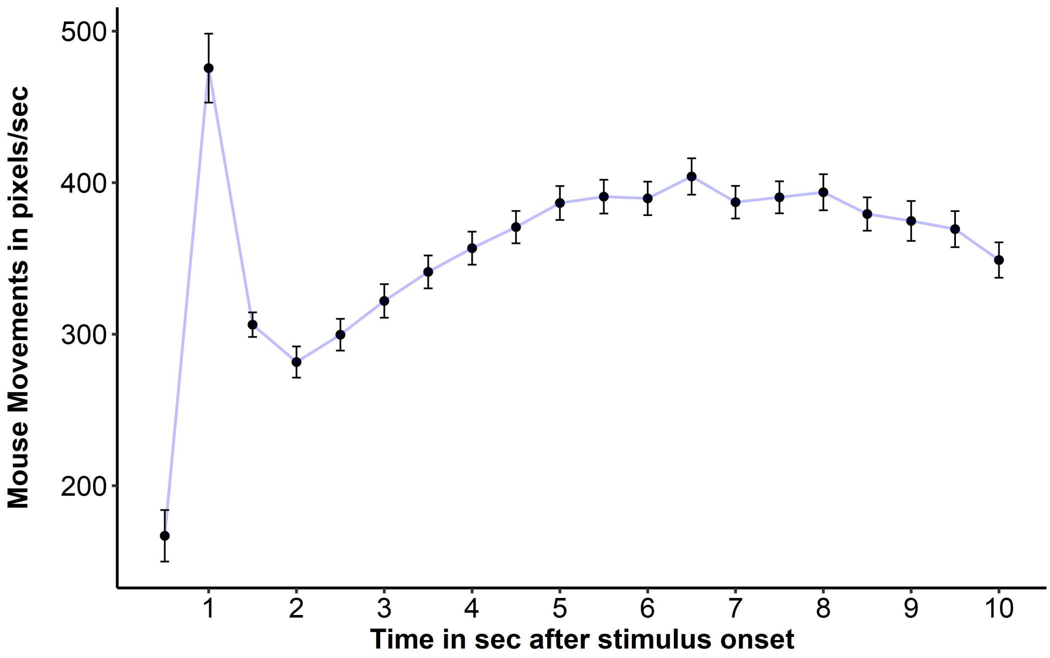

Figure S2: Temporal trajectory of mouse cursor movement throughout the 10 seconds of image presentation. Standard errors represent SEM.

2.3 Distribution of attention towards faces across trials

The percentage of time spent looking at faces (see Figure S3) showed a right-skewed distribution across all trials, with data points located between 1.42 SD below and 6.27 SD above the mean. When log-transforming data after adding 1, all data points were located between 4.32 SD below and 2.93 SD above the mean. Attention towards the face was omitted in 18 out of 2400 trials (0.75%). 13 participants omitted gaze at a face in one trial, one participant omitted gaze at a face in two trials and one participant omitted gaze at a face in three trials.

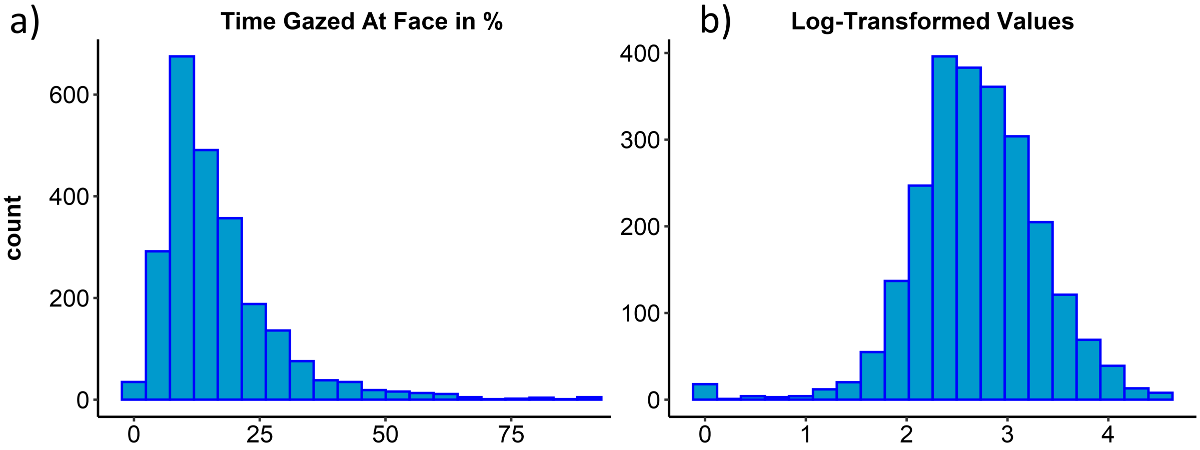

Figure S3: Percentage of time spent looking at faces across all trials (a) and the same data when log-transformed after adding 1 (b). For the purpose of statistical analyses, log-transformed data were used in this study.

2.4 Correlations between attention to faces for images with direct and averted gaze

Table S2: Correlations between attention to faces for images with direct and averted gaze.

	All images			Averted Gaze			Direct Gaze		
	cor	95%-CI	*p*	cor	95%-CI	*p*	cor	95%-CI	*p*
Extraversion	0.21	[0.03, 0.37]	.047	0.15	[-0.03, 0.32]	.199	0.24	[0.07, 0.41]	.024
Agreeableness	0.24	[0.06, 0.40]	.023	0.19	[0.02, 0.36]	.093	0.26	[0.08, 0.42]	.020
Conscientiousness	0.16	[-0.02, 0.33]	.128	0.17	[-0.01, 0.34]	.140	0.14	[-0.04, 0.31]	.178
Neuroticism	-0.16	[-0.33, 0.02]	.128	-0.10	[-0.27, 0.08]	.385	-0.21	[-0.37, -0.03]	.041
Openness	0.24	[0.06, 0.40]	.023	0.23	[0.05, 0.39]	.056	0.22	[0.05, 0.39]	.032
Social Anxiety	-0.26	[-0.42, -0.08]	.023	-0.21	[-0.38, -0.04]	.065	-0.27	[-0.43, -0.10]	.020
Rejection Sensitivity	-0.17	[-0.34, 0.01]	.148	-0.11	[-0.29, 0.07]	.367	-0.20	[-0.37, -0.02]	.087
Trait Anxiety	-0.10	[-0.28, 0.08]	.316	-0.06	[-0.24, 0.12]	.515	-0.13	[-0.30, 0.05]	.178
Depression Levels	-0.24	[-0.40, -0.06]	.023	-0.19	[-0.35, -0.01]	.100	-0.26	[-0.42, -0.08]	.020
Eating Psychopathology	0.04	[-0.14, 0.21]	.736	0.03	[-0.15, 0.21]	.769	0.04	[-0.14, 0.22]	.739
Systemizing	0.01	[-0.17, 0.19]	.876	0.03	[-0.15, 0.21]	.769	0.00	[-0.18, 0.18]	.989
Empathizing	0.24	[0.07, 0.40]	.023	0.23	[0.05, 0.39]	.056	0.23	[0.05, 0.39]	.032
Alexithymia	-0.28	[-0.44, -0.11]	.023	-0.27	[-0.43, -0.10]	.056	-0.26	[-0.42, -0.08]	.031
Impulsivity	-0.08	[-0.25, 0.10]	.463	-0.11	[-0.28, 0.07]	.367	-0.03	[-0.21, 0.15]	.749
Social Value Orientation	-0.09	[-0.27, 0.09]	.398	-0.09	[-0.26, 0.10]	.429	-0.08	[-0.26, 0.10]	.451
General Knowledge	-0.10	[-0.27, 0.08]	.381	-0.10	[-0.27, 0.08]	.384	-0.08	[-0.26, 0.10]	.451
p-Factor	-0.29	[-0.45, -0.12]	.018	-0.24	[-0.41, -0.07]	.056	0.31	[-0.46, -0.14]	.009

2.5 Hierarchical Analysis of Predictors for Preferential Gaze as Faces

Results from a hierarchical analysis testing if any variable predicts attention towards faces beyond personality traits are shown in Table S3. A first all-subsets multiple regression analyses compared the predictive accuracy in models including all combinations of the three personality factors which correlated significantly with attention towards faces (extraversion, agreeableness and openness; models 1 to 8). A second all-subsets multiple regression analyses added every combination of the remaining variables which were correlated significantly with attention towards faces (social anxiety, depression levels, empathizing and alexithymia; models 9 to 23). The best performing models in each step, assessed by means of RMSE in a leave-one-out cross-validation, are printed in bold.

Table S3: Results from a hierarchical analysis testing if any variable predicts attention towards faces beyond personality traits.

Model	Extraversion	Agreeableness	Openness	Social Anxiety	Depression Levels	Empathizing	Alexithymia	RMSE
1								1.0042
2	0.21<br>[0.03, 0.39]							0.9907
3		0.24<br>[0.06, 0.42]						0.9846
4			0.24<br>[0.06, 0.42]					0.9851
5	0.16<br>[-0.02, 0.34]	0.21<br>[0.03, 0.38]						0.9797
6	0.18<br>[0.00, 0.36]		0.22<br>[0.04, 0.39]					0.9787
7		**0.25**<br>**[0.08, 0.42]**	**0.25**<br>**[0.08, 0.42]**					**0.9619**
8	0.13<br>[-0.05, 0.31]	0.22<br>[0.04, 0.40]	0.23<br>[0.06, 0.40]					0.9635
9		**0.22**<br>**[0.05, 0.39]**	**0.24**<br>**[0.07, 0.40]**	**-0.22**<br>**[-0.39, -0.05]**				**0.9422**
10		0.21<br>[0.03, 0.38]	0.24<br>[0.07, 0.41]		-0.18<br>[-0.36, -0.01]			0.9503
11		0.20<br>[0.02, 0.39]	0.22<br>[0.04, 0.40]			0.12<br>[-0.07, 0.31]		0.9636
12		0.19<br>[0.01, 0.37]	0.21<br>[0.04, 0.39]				-0.18<br>[-0.36, 0.00]	0.9561
13		0.21<br>[0.03, 0.38]	0.24<br>[0.07, 0.40]	-0.18<br>[-0.37, 0.01]	-0.10<br>[-0.29, 0.09]			0.9453
14		0.21<br>[0.02, 0.39]	0.23<br>[0.05, 0.40]	-0.21<br>[-0.39, -0.03]		0.04<br>[-0.15, 0.24]		0.9492
15		0.20<br>[0.02, 0.38]	0.22<br>[0.05, 0.39]	-0.18<br>[-0.37, 0.00]			-0.09<br>[-0.30, 0.11]	0.9479
16		0.18<br>[-0.01, 0.37]	0.23<br>[0.05, 0.40]		-0.17<br>[-0.35, 0.01]	0.08<br>[-0.12, 0.27]		0.9560
17		0.18<br>[0.00, 0.36]	0.22<br>[0.05, 0.39]		-0.14<br>[-0.33, 0.05]		-0.12<br>[-0.32, 0.08]	0.9537
18		0.19<br>[0.00, 0.37]	0.21<br>[0.03, 0.39]			0.02<br>[-0.21, 0.25]	-0.17<br>[-0.40, 0.05]	0.9630
19		0.19<br>[0.01, 0.38]	0.23<br>[0.06, 0.40]	-0.17<br>[-0.37, 0.03]	-0.10<br>[-0.29, 0.10]	0.03<br>[-0.16, 0.23]		0.9526
20		0.19<br>[0.01, 0.37]	0.22<br>[0.05, 0.40]	-0.16<br>[-0.36, 0.04]	-0.08<br>[-0.28, 0.12]		-0.07<br>[-0.28, 0.14]	0.9523
21		0.20<br>[0.01, 0.38]	0.22<br>[0.05, 0.39]	-0.18<br>[-0.38, 0.01]		0.00<br>[-0.23, 0.23]	-0.09<br>[-0.33, 0.14]	0.9553
22		0.17<br>[-0.01, 0.36]	0.22<br>[0.04, 0.39]		-0.14<br>[-0.33, 0.05]	0.02<br>[-0.21, 0.25]	-0.11<br>[-0.35, 0.12]	0.9610
23		0.19<br>[0.00, 0.38]	0.22<br>[0.05, 0.40]	-0.16<br>[-0.36, 0.05]	-0.08<br>[-0.29, 0.12]	0.00<br>[-0.23, 0.23]	-0.07<br>[-0.31, 0.17]	0.9600

[a] Values in brackets represent 95%-CI.
